# Risk factors for early and late morbidity in patients with cardiovascular disease undergoing inguinal hernia repair with a tailored approach: a single-center cohort study

**DOI:** 10.1186/s12893-023-01905-y

**Published:** 2023-01-14

**Authors:** Kamran Hajili, Alberto Vega Hernandez, Jakob Otten, Dana Richards, Claudia Rudroff

**Affiliations:** 1 Department of Visceral Surgery and Functional Surgery of the Lower Gastrointestinal Tract (UGI), Evangelisches Klinikum Koeln Weyertal, Weyertal 76, 50931 Cologne, Germany; 2 Department for Cardiology and Intensive Care Medicine, Klinikum Leverkusen, Leverkusen, Germany; 3 Promotion in Medical Studies, Medical Faculty of the University of Cologne, Cologne, Germany

**Keywords:** Cardiovascular disease, Inguinal hernia, Lichtenstein procedure, Postoperative morbidity, Postoperative risk factors, TAPP

## Abstract

**Background:**

Inguinal hernia repair is a common procedure in surgery. Patients with cardiovascular disease have an increased operative risk for postoperative morbidity. The study aimed to identify the most beneficial surgical procedure for these patients.

**Methods:**

Patients undergoing elective surgery for unilateral or bilateral inguinal hernia between December 2015 and February 2020 were included. The cohort was divided into the group of patients with (CVD group) and without (NO group) cardiovascular disease and analyzed according to the postoperative morbidity distribution and correlated to the surgical technique used.

**Results:**

Of the 474 patients included 223 (47%) were operated on using the Lichtenstein technique and 251 (53%) using TAPP, respectively. In the CVD group the Lichtenstein procedure was more common (n = 102, 68.9%), in the NO group it was TAPP (n = 205, 62.9%; p < 0.001). 13 (8.8%) patients in the CVD group and 12 (3.7%) patients in the NO group developed a postoperative hematoma (p = 0.023). In the further subgroup analysis within the CVD group revealed cumarine treatment as a risk factor for postoperative hematoma development, whereas the laparoscopic approach did not elevate the morbidity risk.

**Conclusion:**

CVD is a known risk factor for perioperative morbidity in general surgery, however, the TAPP method does not elevate the individual perioperative risk.

## Introduction

Inguinal hernia repair is one of the most common surgeries in the world. In Germany 275,000 inguinal hernia repairs are performed every year [1], with the Lichtenstein procedure being the most common surgical method [2]. Postoperative complications generally affect the recovery process and quality of life of the patients concerned [3].

Patients with cardiovascular disease (CVD) undergoing surgery are at a substantial risk of morbidity due in part to their need for antithrombotic medication. Complications during or after surgery can further impair their well-being.

This observational study analyzed the outcomes of inguinal hernia procedures with special attention to the subgroup of patients with CVD. The aim of the study was to identify the most common morbidities that affect the outcome of the surgical procedure and the subsequent course of CVD, as well as the most beneficial method of inguinal hernia repair for these patients to avoid morbidity.

## Patients and methods

### Inclusion criteria and informed consent

Between December 2015 and February 2020, all consecutive patients undergoing elective surgery for unilateral or bilateral inguinal hernia at Evangelisches Klinikum Koeln Weyertal were included. Before surgery, patients gave their informed consent to participate in the Herniamed Registry [4]. We confirm that the study has been performed in accordance with the Declaration of Helsinki and all methods were performed in accordance with the relevant guidelines and regulations.

### Data collection

For all patients, data on characteristics such as sex, age, surgical procedure, body mass index (BMI), severity of preoperative comorbidity according to the classification of the American Society of Anesthesiologists (ASA) [5], surgical outcome including operative time, surgical procedure, morbidity and mortality, postoperative pain scale according to the Numeric Rating Scale (NRS) on the day after surgery and upon discharge, and the length of hospital stay were collected prospectively in the Herniamed database.

Missing data and details on antithrombotic medication and comorbidity were completed from hospital records. In cases of antiplatelet treatment the medication was continued, whereas the application of novel oral anticoagulants (NOACs) was paused 24 h prior to surgery and bridged with low molecular weight heparins until discharge [6]. Cumarines were paused 10 days prior to surgery, bridged with low molecular weight heparins and restarted 10 days after surgery. All patients with antithrombotic mediation were operated under general anesthesia. Regional anesthesia was used in some cases, whereas local anesthesia is generally not performed in our setting. No outpatient surgery was planned in our setting.

### Morbidity and mortality assessment

Morbidity and mortality were rated according to the Clavien Dindo Classification (CDC), ranging from 0 to 5 [7]. Minor morbidities assumed CDC scores from 1 to 3a, whereas major morbidities assumed CDC scores from 3b to 5. Early morbidities referred to all events within 30 days of surgery. In case of swelling, local discomfort, or discoloration in the inguinal region clinical examination and ultrasound were performed by the surgeon in charge to identify wound infection and/or hematoma.

### Follow up

Follow-up results were obtained based on responses to letters sent to all patients 12–24 months after their surgery. The questionnaire asked for symptoms of pain ≥ 4 according to the NRS, dysesthesias, reoperation on the hernia site, recurrences, late hematoma, and infection that occurred after discharge from the hospital. Patients with complaints were offered a consultation. In cases of unopened letters returned to sender further inquiries to locate the patients were undertaken. In 58 cases no valid address could be identified, and the respective patients were lost to follow up. Unanswered letters were resent a second time. In cases of unanswered letters the general practitioner was contacted but did not provide detailed information on the Herniamed questionnaire.

### Subgroup definition and data analysis

The cohort was analyzed in two groups, patients with (CVD group) and without cardiovascular disease (NO group). The CVD group comprised patients with coronary heart disease (CHD), previous myocardial infarction (MI), atrial fibrillation (AF), congestive heart disease (CON), arterial hypertension (AH), and/or apoplexy (APO), with patients suffering from one to five of the conditions mentioned above.

The results from the CVD group and the NO group were further analyzed to determine the correlation of morbidity with the surgical technique used.

Data were analyzed using the SPSS statistical package (version 27.0.0.0, IBM). Distributions of quantitative variables are presented as the mean (± standard deviation [SD]) and analyzed by the Mann–Whitney-U test. Multiple testing was adjusted with a post hoc test for 2 × 3 and more variables. Qualitative variables are summarized as count, percentage, median, and range and were compared using the chi-square test. A two-sided p value of < 0.05 was considered statistically significant.

## Results

### Baseline characteristics

Altogether 474 patients were included in this study, of whom 148 (31%) suffered from CVD. In the CVD group, 36 (18.6%) suffered from CHD, 21 (14%) had previous MI, 10 (14%) AF, 56 (38%) CON, 130 (87%) AH, and 5 (3%) APO. [8] See also Table 1.

**Table 1:** Stratification of all 148 patients with cardiovascular into individual diagnoses

Cardiovascular disease	n = 148
Coronary Heart Disease	36 (24%)
Myocardial Infarction	21 (14%)
Atrial Fibrillation	10 (7%)
Congestive Heart Disease	56 (38%)
Arterial Hypertension	130 (87%)
Apoplexy	5 (3%)

Data are presented as total number and percentages (%) with respect to the CVD subgroup

One patient suffered from 5 conditions, 18 patients from 4 conditions, 16 patients from 3 conditions, 29 patients from 2, and 82 patients from one condition. The average CVD patient suffered from 1.76 cardiovascular conditions. One patient suffered from 5 conditions, 18 patients from 4 conditions, 16 patients from 3 conditions, 29 patients from 2, and 82 patients from one condition. The average CVD patient suffered from 1.76 cardiovascular conditions

Patients in the total cohort were between 18 and 102 years old (median: 57 years, IQR: 25) on the day of surgery. Patients in the CVD group were significantly older than those in the NO group, (CVD [median: 71, IQR: 18, range 22–102] and NO [median: 53, IQR: 19, range 18–87]; p < 0.005). Of the 474 patients, 388 (81.9%) were male. The distribution of the sexes in the subgroups (CVD and NO) was comparable. Comorbidity was expressed by the American Society of Anesthesiologists Classification (ASA) [9].For the total cohort 29 (6.2%) patients were ASA III, no patient was ASA IV or ASA V. Comorbidity was significantly higher in the CVD group than in the NO group, (CVD [n = 20, 14.1%] and NO [n = 9, 2.8%]; p < 0.001). The mean BMI was 25.3 kg/m^2^ (range = 16.3–53, IQR: 4, SD = 3.9) with no variation between the groups.

Altogether 49 patients received antithrombotic therapy, all of whom were within the CVD group. The majority of patients received antiplatelet medication (n = 39), six patients NOACs, and four patients cumarines. For further details with respect to the CVD diagnosis and antithrombotic therapy see Table 2.

**Table 2:** Stratification of the diagnosis with the antithrombotic agent for the 49 patients in the CVD subgroup with antithrombotic treatment

	Antiplatelet	NOAC	Cumarine
	n = 39	n = 6	n = 4
CVD			
Coronary heart disease	27	3	4
Myocardial infarction	15	2	3
Arterial fibrillation	1	6	3
Congestive heart disease	24	5	4
Arterial hypertension	31	2	2
Apoplexy	2	1	0

The laparoscopic transperitoneal preperitoneal hernia repair (TAPP) and Lichtenstein procedure were distributed almost equally in the overall cohort, i.e. 251 (53%) patients were operated in TAPP technique and 223 (47%) performing the Lichtenstein procedure. The most frequently used method in the CVD group was the Lichtenstein procedure, (Lichtenstein [n = 102, 68.9%] and TAPP [n = 46, 31.1%]), which differed significantly from the NO group, where the TAPP technique was more common, (Lichtenstein [n = 121, 37.1%] and TAPP [n = 205, 62.9%]; p < 0.001).

Within the group of patients with antithrombotic treatment the TAPP procedure was solely performed under antiplatelet therapy in three cases. All other patients underwent the Lichtenstein procedure.

All laparoscopic procedures and most of the open procedures were performed in general anesthesia (n = 468, 98.7%). In 6 (1.3%) cases a regional anesthesia was applied, all within the NO group. No local anesthesia was used in our setting.

The median operative time for all patients was 70.9 min (range: 20–212 min), with no significant differences between the groups (p = 0.544).

The mean length of hospital stay was 3 days (range: 0–41 days), with no difference between the groups (p = 0.207). Although no day surgery was scheduled in our setting, altogether 10 patients, 3 from the CVD group and 7 from the NO group, left the hospital on the day of surgery on their own behalf.

All results are summarized in Table 3.

**Table 3:** Patients’ characteristics, surgical technique, and perioperative results for the total cohort, the subgroup with cardiovascular disease (CVD), and the subgroup without cardiovascular conditions (NO)

	All patients n = 474	CVD n = 148	NO n = 326	p
Age, median (range)	58 (24)	70 (18)	52 (19)	< 0.001
Male, n (%)	388 (81.9%)	124 (83.8%)	264 (81%)	0.463
Female, n (%)	86 (18.1%)	24 (16.2%)	62 (19%)	
ASA, n (%)				< 0.001
1	8 (1.7%)	1 (0.7%)	7 (2.2%)	
2	430 (92.1%)	121 (85.2%)	309 (95.1%)	
3	29 (6.2%)	20 (14.1%)	9 (2.8%)	
4	0	0	0	
5	0	0	0	
BMI, mean (range)	25.3 (16.3–53)	26.1 (17–53)	24.9 (16.3–41)	0.005
Antithrombotic treatment	49 (10.3%)	49 (33.1%)	0	< 0.001
Lichtenstein n (%)	223 (47%)	102 (68.9%)	121 (37.1%)	< 0.001
TAPP n (%)	251 (53%)	46 (31.1%)	205 (62.9%)	
Anesthesia				
General, n (%)	468 (98.7%)	148 (100%)	320 (98.2%)	0.033
Regional, n (%)	6 (1.3%)	0	6 (1.8%)	
Local	0	0	0	
Operating time (min), median (range)	70.9 (20–212)	69.9 (25–210)	71.3 (20–212)	0.544
Days of hospital stay, mean (range)	3 (0–41)	3 (0–17)	3 (0–42)	0.021

Data are presented as the median (age and duration of hospital stay) or the mean (BMI and operating time) and their range for continuous variables and as total number and percentages (%) for binary variables. The p-value indicates the level of statistical significance.

### Morbidity and mortality

Complications after surgery occurred in 81 (17.2%) patients (CVD [n = 29, 19.6%] and NO [n = 52, 16.1%]; p = 0.358). 65 (13.8%) had minor complications (CDC 1–3a) and 16 (3.4%) had major complications (CDC 3b–5). No significant difference (p = 0.385) was observed in terms of morbidity distribution between the two groups.

An average of 1.31 complications were observed with one patient suffering from four complications, 3 patients from 3 complications, 16 patients from two complications and 61 patients from one complication.

A postoperative hematoma occurred in 25 (5.3%) patients and significantly more often in patients within the CVD group (CVD [n = 13, 8.8%] and NO [n = 12, 3.7%]; p = 0.023). A detailed subgroup analysis is listed further below.

Early recurrence occurred in eight patients with no differences between the two groups, (CVD [n = 3, 2%] and NO [n = 5, 1.6%]; p = 0.712).

Postoperative pain of high intensity (≥ 4 on the NRS) on day 1 after surgery was noted in 53 (12.8%) patients (CVD [n = 16, 12.6%] and NO [n = 37, 12.9%]; p = 0.924) and at discharge in 10 (2.5%) patients (CVD [n = 5, 3.9%] and NO [n = 5, 1.8%]; p = 0.208) with no significant difference between the subgroups. For details on morbidity see Table 4.

**Table 4:** Postoperative morbidity and detailed distribution according to Clavien Dindo classification (CDC), stratified into minor and major morbidity

	All patients n = 474	CVD n = 148	No n = 326	p
Morbidity, n (%)	81 (17.2%)	29 (19.6%)	52 (16.1%)	0.358
CDC, n (%)				
CDC minor	65 (80.3%)	22 (75.9%)	43 (82.7%)	0.490
CDC major	16 (19.7%)	7 (24.1%)	9 (17.3%)	
0	389 (82.8%)	119 (80.4%)	270 (83.9%)	0.481
1	48 (10.2%)	18 (12.2%)	30 (9.3%)	
2	10 (2.1%)	2 (1.4%)	8 (2.5%)	
3a	7 (1.5%)	2 (1.4%)	5 (1.6%)	
3b	16 (3.4%)	7 (4.7%)	9 (4.7%)	
4	0	0	0	
5	0	0	0	
Postoperative hematoma, n (%)	25(5.3%)	13 (8.8%)	12 (3.7%)	0.023
Early recurrence, n (%)	8 (1.7%)	3 (2%)	5 (1.5%)	0.712
Postoperative Pain ≥ 4 NRS Day 1, n (%)	53 (12.8%)	16 (12.6%)	37 (12.9%)	0.924
Postoperative pain ≥ 4 NRS, n (%)	10 (2.5%)	5 (3.9%)	5 (1.8%)	0.208

Data are presented as total number and percentages (%) for binary variables. The p-value indicates the level of statistical significance

Further details on the main morbidities are listed and distributed according to the subgroups

### Subgroup analysis on postoperative hematoma

Patients operated on by the Lichtenstein method experienced significantly more hematomas than those operated on in TAPP technique (Lichtenstein [n = 17, 7.7%], TAPP [n = 8, 3.2%]; p = 0.033).

Within the CVD subgroup 12 patients experienced a hematoma (3.7%), 6 (50% of the CVD group, 12.2% within the group of antithrombotic treated patients) of whom were under antithrombotic therapy, whereas 43 (87.8%) patients with antithrombotic therapy did not develop a hematoma. Of the six patients with antithrombotic therapy who developed a postoperative hematoma all underwent the Lichtenstein procedure.

See Table 5.

**Table 5:** Stratification postoperative hematoma and antithrombotic therapy with respect to the surgical procedure

Antithrombotic therapy	All	No	Yes	p
Hematoma vs surgical procedure	25 (5.3%)	19 (4.5%)	6 (12.2%)	0.033
Hematoma Lichtenstein, n (%)	17(7.7%)	11 (57.9%)	6 (100%)	
Hematoma TAPP, n (%)	8 (3.2%)	8 (42.1%)	0	

Data are presented as total number and percentages (%) for binary variables. The p-value indicates the level of statistical significance

Of the six patients with antithrombotic therapy who developed a postoperative hematoma three had cumarine medication, one received NOACs, and two patients were under antiplatelet treatment. The post hoc testing of hematoma versus antithrombotic treatment revealed a significance risk for cumarine medication only. See Table 6.

**Table 6:** Stratification of the diagnosis with the antithrombotic agent for the 49 patients in the CVD subgroup with antithrombotic treatment

	Antiplatelet	NOAC	Cumarine	*p*
	n = 39	n = 6	n = 4	
Postoperative Hematoma				< 0.001
No	37 (94%)	5 (83.3%)	1 (25%)	
Yes	2 (5.1%)	1 (16.7%)	3 (75%)	

Data are presented as total number and percentages (%) for binary variables. The p-value indicates the level of statistical significance. After post-hoc test for postoperative hematoma against type of anticoagulation significance after Bonferoni correction is shown only for the column cumarine with a Z-value of 4. At a chi^2^ of p = 0.0003417 corrected by 0.008333333 and at a significance level of 0.05 results in p = 0.0020502, thus the event is significant

### Follow-up

Of the 474 follow-up letters sent to the patients 12–24 months after surgery, 185 (39%) completed letters were returned. In total, 52 (28.1%) patients remarked late morbidity after surgery with an average of 1.1 complaints for the cohort and 7 patients with two different complications. There was no difference between the subgroups.

Dysesthesia was documented in 27 (14.6%) patients (CVD [n = 11, 15.9%] and NO [n = 16, 13.8%]).

Late recurrence was seen in 5 (2.7%) patients in the total cohort (CVD [n = 1, 1.4%] and NO [n = 4, 3.4%]).

Chronic pain ≥ 4 NRS after discharge from the hospital was reported in 18 (9.7%) patients (CVD [n = 5, 7.2%] and NO [n = 13, 11.2%]).

Seven patients (3.7%) reported a hematoma (CVD [n = 2, 2.9%] and NO [n = 5, 4.3%]) and two patients (1.1%) complained about a wound infection after discharge from the hospital (CVD [n = 1, 1.4%] and NO [n = 1, 0.9%]). See Table 7.

**Table 7:** Patients’ follow-up data with morbidity and main complaints

	All patients n = 185	CVD group n = 69	NO group n = 116	p
Morbidity, n (%)	52 (24.6%)	18 (26.1%)	34 (29.3%)	0.623
Dysesthesia, n (%)	27 (14.6%)	11 (15.9%)	15 (13.8%)	
Recurrence, n%	5 (2.7%)	1 (1.4%)	4 (3.4%)	
Chronic pain ≥ 4 (NRS) follow up, n (%, N)	18 (9.7%)	5 (7.2%)	13 (11.2%)	
Late Hematoma, n (%)	7 (3.7%)	2 (2.9%)	5 (4.3%)	
Late Wound Infection, n (%)	2 (1.1%)	1 (1.4%)	1 (0.9%)	

Data are presented as total number and percentages (%). The p-value indicates no statistical significant difference for the overall morbidity. No further testing was performed due to the complex cross table and the little incidences in the individual complaints

## Discussion

This present study demonstrates an increased perioperative complication risk, particularly for the development of hematoma, for patients with a cardiovascular risk profile. The results confirm the result from Turrentine and colleagues on the elevated risk of congestive heart disease for morbidity and reoperation in non-cardiac surgery [10] and a great nationwide population-based cohort study from Taiwan in 2017 by Lee and colleagues, who identified increased perioperative morbidity for hernia procedures in patients with previous cardiovascular disease compared to patients with no such risk [11].

Thereby, the subgroup analysis showed that especially patients with antithrombotic treatment have an increased risk for the formation of a hematoma. In the further post hoc analysis, the study identified the increased risk mainly for patients with cumarine therapy, but not for the patients with antiplatelet medication and with NOACs.

Interestingly, it was even shown that the group of patients on antiplatelet drugs for antithrombotic treatment did not show an increased risk profile compared to the overall collective. This again emphasizes that antiplatelet medication does not need to be paused for patients undergoing general surgery, in this case inguinal hernia surgery. This has also been shown in several previous studies and reviews [12–14]. Surprisingly, this was also shown for patients on NOACs in our study. However, for this subgroup, the small number of patients being treated limits the significance, and statistical validity is not given. Only cumarine therapy, which has been eclipsed by current and modern antithrombotic therapy methods, still has a significantly increased risk of a bleeding complication. These results are also confirmed in other studies as by Zeb and colleagues in 2016 [15].

The safety of the surgical procedure choice is also unaffected by the antithrombotic treatment modality for antiplatelet agents and NOACs, but not for cumarines. However, the validity of the results is limited by the fact that the surgeries performed in patients with NOACs and cumarines were all with the Lichtenstein procedure. This practice stems from the still-valid "informal" recommendation that patients with higher risk profiles are better served by an open surgical procedure than by laparoscopic surgery [16, 17], which has already been shown invalid with respect to wound hematoma and infection in an updated network meta-analysis of randomized trials by Aiolfi and colleagues in 2021 [18]. And although the patients with NOAC therapy did not show an increased risk of bleeding in our setting, a conclusion on the risk with laparoscopic surgery from our data is not valid.

With regard to the surgical procedure the subgroup of patients on antiplatelet drugs, however, showed no difference in perioperative morbidity compared with the overall population. This conclusion is also reached by other studies, which do not find any relevant risk constellation due to antiplatelet therapy for the laparoscopic procedure. For example, Hill and colleagues published in 2019 that the laparoscopic procedure under antiplatelet therapy does not have a relevant, higher risk profile than open surgery [19]. Particularly regarding older patients on antithrombotic therapy, Hada and colleagues were able to show it too, there is no increase in the risk of postoperative complications [20]. And even though Staerkle and colleagues found an increased risk for bleeding complications under antithrombotic therapy according to data from the Herniamed registry, the complication-related reoperations seemed to be lower in the laparoscopic approach [21], which as once more confirmed by Köckerling and colleagues, as well [22]. Thus, at least from a medical point of view, the laparoscopic procedure is not riskier than the open approach, even in patients with an increased cardiovascular risk profile and antithrombotic treatment as also previously stated by Ho and colleagues in 2019 [23] and if current perioperative risk management patterns are followed, as stated by Balch and colleagues in 2022 [17]. It is high time to reconsider the paradigm—that the open procedures in hernia surgery are safer—and to evaluate it conclusively with studies on this topic.

Further analysis of the collective showed that the group of patients with cardiovascular risk profile had a higher comorbidity in the ASA classification, a higher age and as a further risk factor a higher BMI. Thus, with increasing age and other risk factors, the risk of cardiovascular disease also increases; this is a finding that confirms common knowledge.

Furthermore, the inpatient length of stay stands out in the study. In this setting, patients underwent surgery were exclusively scheduled under inpatient conditions, with an average hospital length of stay of three days. The duration did not differ between the overall collective and patients with or without a cardiovascular risk profile. And although a small subgroup (n = 10) left the hospital on the day of surgery; this was not planned but at the expressive request of the individual patient. This subgroup also included three patients with cardiovascular risk profiles.

In any case, the inpatient form of treatment and the duration of treatment cannot be used to derive a statement about the complication prevalence or the risk of the intervention.

This is rather a German specialty. Surgical treatment of inguinal hernias is still a mostly inpatient business in Germany, which incidentally has the lowest rate of outpatient and day surgery, not only for inguinal hernia surgery. In this respect Germany differs significantly from all other countries in Europe. On the one hand this is due to the cultural and structural orientation of the health care system toward a high density of hospital beds, and on the other hand to the lack of incentive for day surgery and the lack of structural infrastructure in the outpatient setting [24]. The political and health insurance problems resulting from this circumstance have been recently thoroughly investigated and addressed by Albrecht and colleagues [25]. The length of inpatient stay in this study should therefore not be regarded as a medical statement, but as a cultural feature.

Standardized letters were sent on the follow-up data in a time window up to 24 months after the intervention. Since in many cases the recipients had moved or could no longer be found, and many patients had certainly been away from the procedure for a very long time, the response rate of the questionnaires (38%) was disappointing, although it is in line with published data on response rates of postal surveys, as published previously by Coughlin and colleagues and Anhang Price and colleagues [26]. This limits the validity of the follow-up data. These results did not differ from the overall collective in the risk group with cardiovascular risk profile, though. In this respect, they have limited usability and are also of little significance.

## Conclusion

The results of the study confirm the increased risk of surgical procedures for patients with cardiovascular risk profiles, especially regarding postoperative hematomas. However, this was mainly the case for patients on antithrombotic treatment and especially cumarine therapy.

At the same time, the results confirmed the safety of laparoscopic hernia interventions also for patients on antiplatelet drugs. Considering the fact that especially bilateral hernias can be treated simultaneously and without problems, this procedure should be given a higher priority also for patients with cardiovascular risk profile. For this purpose, patients with and without cardiovascular risk profile should be included in a prospective randomized multicenter study to confirm safety and feasibility without further medical risk.

## Data Availability

All data collected and analyzed during the study are available upon request.

## Abbreviations

CVD: Cardiovascular disease
MI: Myocardial infarction
CHD: Coronary heart disease
AH: Arterial hypertension
CON: Congestive heart disease
AF: Arterial fibrillation
APO: Apoplexy
TAPP: Laparoscopic transabdominal preperitoneal patch
NRS: Numeric rating scale
BMI: Body mass index
CDC: Clavien Dindo Classification
SD: Standard deviation
IQR: Interquartile range
